# Carbon Dots @ Platinum Porphyrin Composite as Theranostic Nanoagent for Efficient Photodynamic Cancer Therapy

**DOI:** 10.1186/s11671-018-2761-5

**Published:** 2018-11-08

**Authors:** Fengshou Wu, Liangliang Yue, Huifang Su, Kai Wang, Lixia Yang, Xunjin Zhu

**Affiliations:** 1 Key Laboratory for Green Chemical Process of the Ministry of Education, School of Chemical Engineering and Pharmacy, Wuhan Institute of Technology, Wuhan, 430205 People’s Republic of China; 2 Department of Orthopaedics, The First Affiliated Hospital of Zhengzhou University, Zhengzhou, 450052 People’s Republic of China; 3 Department of Chemistry, Hong Kong Baptist University, Waterloo Road, Hong Kong, People’s Republic of China

**Keywords:** Carbon dots, Porphyrin, Photodynamic therapy, Energy transfer, Photoluminescence

## Abstract

Photosensitizers are light-sensitive molecules that are highly hydrophobic, which poses a challenge to their use for photodynamic therapy. Hence, considerable efforts have been made to develop carriers for the delivery of PSs. Herein, we synthesized a new theranostic nanoagent (CQDs@PtPor) through the electrostatic interaction between the tetraplatinated porphyrin complex (PtPor) and the negatively charged CQDs. The size and morphology of as-prepared CQDs and CQDs@PtPor were characterized by a series of methods, such as XRD, TEM, XPS, and FTIR spectroscopy. The CQDs@PtPor composite integrates the optical properties of CQDs and the anticancer function of porphyrin into a single unit. The spectral results suggested the effective resonance energy transfer from CQDs to PtPor in the CQDs@PtPor composite. Impressively, the CQDs@PtPor composite showed the stronger PDT effect than that of organic molecular PtPor, suggesting that CQDs@PtPor is advantageous over the conventional formulation, attributable to the enhanced efficiency of ^1^O_2_ production of PtPor by CQDs. Thus, this CQDs-based drug nanocarrier exhibited enhanced tumor-inhibition efficacy as well as low side effects in vitro, showing significant application potential in the cancer therapy.

**Electronic supplementary material:**

The online version of this article (10.1186/s11671-018-2761-5) contains supplementary material, which is available to authorized users.

## Background

Photodynamic therapy (PDT) has been widely practiced as a promising non-invasive therapeutic modality for the treatment of many human diseases including several conditions of the skin, age-related macular degradation, and cancer [1]. PDT can be used alone or in combination with surgery, chemotherapy, or ionizing radiation [2]. In photodynamic therapy, photosensitizers (PSs) are irradiated by a specific wavelength of light, which triggers the generation of reactive oxygen species from intracellular oxygen that consequently induce cell death and necrosis of proximal tissues [3–6]. Because photosensitizers are typically harmless without light, tumor treatment can be precisely targeted by selective illumination, thus limiting damage to surrounding healthy tissues [7–9]. Activatable photosensitizers, such as porphyrin and phthalocyanines derivatives, have been demonstrated to possess simultaneous cancer imaging and therapy capabilities, and some of these photosensitizers have been approved for clinical use [10, 11]. However, many of them are limited because of poor water solubility, prolonged cutaneous photosensitivity, inadequate selectivity, and their inability to be absorbed in the region (> 700 nm) where the skin is most transparent, which are encountered in clinical applications of numerous traditional chemicals. Therefore, numerous approaches have been proposed to incorporate PSs into carriers such as liposomes [12], polymeric nanoparticles [13, 14], gold nanoparticles [15–17], carbon nanotubes [18], graphenes [19], and carbon nanodots [20–22].

Recently, carbon quantum dots (CQDs), as a new type of carbon nanomaterial, have attracted considerable attention owing to their unique properties, such as superior optical properties, excellent water solubility, low toxicity, excellent biocompatibility, good cell permeability, and facile preparation and modification. Thus, CQDs have been demonstrated many promising applications in optoelectronics, sensing [23, 24], theranostic [25–27], and bioimaging fields. During the past few years, numerous methods have been developed for synthesizing a variety of CQDs, such as hydrothermal method, microwave method, thermal treatment method, and electrochemical method [28]. Among them, hydrothermal methods using natural precursors to produce CQDs have been widely reported due to their green chemistry nature [29, 30].

Moreover, CQDs has the potential to be a loading platform for various molecules due to their abundant surface groups and reasonable biocompatibility [31, 32]. In particular, when functionalized with different chemical groups, CQDs can be engineered with various functional elements such as drug molecules, protein, and aptamer by covalent or noncovalent interaction for versatile biomedical applications [33]. For example, in 2012, Huang et al. designed a novel theranostic platform based on photosensitizer-conjugated carbon dots. Upon irradiation, the prepared CQDs-Ce6 displayed the stronger fluorescence emission and higher photodynamic efficacy relative to Ce6 alone [34]. In 2014, Choi et al. developed a similar theranostic platform based on FA-conjugated CQDs loaded with ZnPc [3]. In the same year, Wang et al. developed conjugates by electrostatically connecting TMPyP with non-toxic CQDs [35]. In 2015, Beack et al. synthesized a CQDs-Ce6-HA conjugate, which showed much higher photodynamic effect than that of free Ce6 and CQDs-Ce6 [36].

**Scheme 1:**
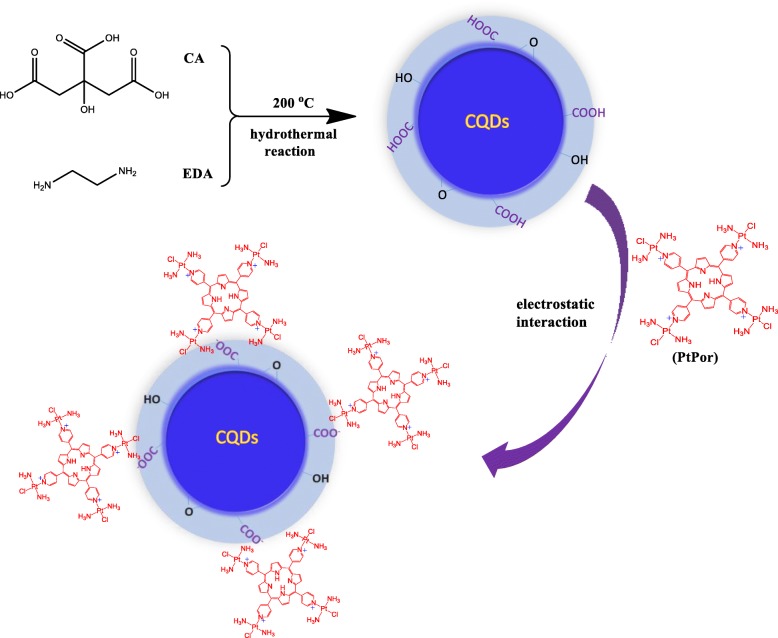
Schematic illustration of preparation of CQDs@PtPor

More recently, a new tetraplatinated porphyrin complex was reported by Naik et al. The results showed that the platinum porphyrin displayed minor cytotoxicity in the dark, but IC_50_ values down to 19 nM upon 420-nm laser irradiation, suggesting that the tetraplatinated porphyrin complex is a promising anticancer agent for cancer therapy [37]. However, the synthesized tetraplatinated porphyrins exhibited low biocompatibility and water solubility, which limited their clinical use. To this end, here, we develop a new theranostic nanoagent (CQDs@PtPor) through the electrostatic interaction between tetraplatinated porphyrin complex (PtPor) and the negatively charged CQDs (Scheme 1). The CQDs@PtPor composite integrates the optical properties of CQDs and the anticancer function of porphyrin into a single unit. The spectral results suggested the effective resonance energy transfer from CQDs to PtPor in the CQDs@PtPor composite. Impressively, the CQDs@PtPor showed the stronger PDT effect than that of PtPor alone, which might be assigned to the higher efficiency of ^1^O_2_ generation of PtPor by CQDs. Moreover, small size of CQDs@PtPor might enable to selective accumulation in tumor site through the EPR effect. Thus, the as-prepared nanoagnet (CQDs@PtPor) showed great application potential in the cancer therapy.

## Methods

Trans-platinum diammine dichloride (transplatin) was purchased from Aladdin®. 1,3-Diphenylisobenzofuran (DPBF) was obtained from Sigma-Aldrich. All the solvents were purchased from Tianjin Fu Chen Chemical Reagents. The other chemicals were purchased from Sinopharm Chemical Reagent Co., Ltd. and used as received.

### Synthesis of [Trans-PtCl(NH_3_)_2_]_4_-5,10,15,20-Tetra(4-pyridyl)-Porphyrin Nitrate

Transplatin (0.193 mmol, 58 mg) and silver nitrate (0.193 mmol, 33 mg) were dissolved in 5 mL of DMF. After string for 24 h, the white silver chloride formed was removed from the resulted turbid solution through the centrifugation to acquire the clear solution, which was then added to the suspension of 5,10,15,20-Tetra(4-pyridyl)porphyrin (0.487 mmol, 30 mg) in 3 mL DMF. After stirring at 50 °C for 48 h, the mixture was cooled down to room temperature. Then, 10 mL diethyl ether was added to get the red precipitate, which was then washed with methanol, dichloromethane, and diethylether. Finally, the sample was dried under vacuum to acquire 81 mg product. Yield 86%. ^1^H NMR (400 MHz; DMSO-d_6_): δ 9.45 (d, 8H), 9.14 (s, 8H), 8.52 (m-pyridyl, d, 8H), 4.70 (NH_3_, s, 24H), − 3.04 (s, 2H); MS (ESI): *m*/*z* = 1209 [M-3(NO_3_)-2{PtCl(NH_3_)_2_}]^+^, 1074 [M-4(NO_3_)-2{PtCl(NH_3_)_2_}-2NH_3_-Cl-2H]^+^, 883 [M-4(NO_3_)-3{PtCl(NH_3_)_2_}]^+^, 866 [M-4(NO_3_)-3{PtCl(NH_3_)_2_}-NH_3_]^+^, 812 [M-4(NO_3_)-3{PtCl(NH_3_)_2_}-Cl-2(NH_3_)]^+^, 574 [M-4(NO_3_)-2{PtCl(NH_3_)_2_}]^2+^.

### Preparation of the CQDs

Generally, citric acid (0.45 g) and ethylenediamine (500 μL) was dissolved in DI-water (10 mL). Then the solution was transferred to a poly (tetrafluoroethylene) (Teflon)-lined autoclave (30 mL) and heated at 200 °C for 5 h. After the reaction, the reactors were cooled to room temperature by water or naturally. The crude product, which was brown-black, was purified in a centrifuge for 30 min to remove agglomerated particles, and then dialyzed against DI water to obtain the CDs.

### Preparation of the CQDs@PtPor composite

The PtPor molecule, bearing four positive charges in pyridine ring, can bind on the surfaces of the negatively charged CQDs through an electrostatic interaction to obtain the CQDs@PtPor composite. In general, 20 mg PtPor dissolved in 3 mL DMSO was dispersed in 12 mL water. The solution was added slowly into the CQDs suspension (5 mg CQDs dissolved in 15 mL H_2_O) under sonication. After stirring at room temperature for 24 h, the solution was purified in a centrifuge for 30 min to remove agglomerated particles, and then dialyzed against DI water for 2 days. The aqueous solution of CQDs@PtPor was lyophilized at 4 °C to yield the desired product.

### The Calculation of Quantum Yields of CQDs

The quantum yield of CQDs was measured with quinine sulfate as the reference (0.1 M H_2_SO_4_ aqueous solution, fluorescent quantum yield ∼ 54%) by the following equation:

$$ \upvarphi \kern0.5em =\kern0.5em {\upvarphi}_{\mathrm{st}}\left(I/{I}_{\mathrm{st}}\right)\;{\left(\upeta /{\upeta}_{\mathrm{st}}\right)}^2 $$

Where Φ is fuorescence quantum yield, *I* is the slope of curves, and η is the refractive index of solvent. The subscript “st” refers to the reference of known quantum yield (quinine sulfate in 0.1 M H_2_SO_4_). The absorption was kept below 0.1 at the excitation wavelength of 360 nm to minimize reabsorption.

#### Singlet Oxygen Generation

A solution of the sample and 3-diphenylisobenzofuran were irradiated in a glass cuvette (3 mL), at room temperature. The absorption decay of DPBF at 415 nm was measured at irradiation intervals of 3 min up to 30 min. The production of singlet oxygen was evaluated qualitatively through the DPBF, a singlet oxygen quencher. The percentage of the DPBF absorption decay, proportional to the production of ^1^O_2_, was assessed by the difference between the initial absorbance and the absorbance after a given period of irradiation. Each experiment was repeated three times.

#### Cytotoxicity Assay of CQDs, PtPor, and CQDs@PtPor

Human cervical carcinoma (HeLa) cells were cultured in Dulbecco’s modified Eagle’s medium (DMEM) supplemented with 5% fetal calf serum (FCS), 100 U/mL penicillin, 100 μg/mL streptomycin at 37 °C, and 6% CO_2_. The methylthiazolyltetrazolium (MTT) viability assay was performed according to a standard method. In brief, HeLa cells (3 × 10^3^/well) were seeded in 96-well plates for 24 h prior to exposure to drugs. The cells were treated with samples overnight in the dark. The cytotoxicity was determined by the MTT reduction assay. The cell monolayers were rinsed twice with phosphate-buffered saline (PBS) and then incubated with 50 μL MTT solution (0.5 mg/mL) at 37 °C for 3 h. After the media were removed, 100 μL of DMSO was added. The solution was shook for 30 min to dissolve the formed formazan crystals in living cells. The absorbance was measured at dual wavelength, 540 nm and 690 nm, on a Labsystem Multiskan microplate reader (Merck Eurolab, Switzerland). Each dosed concentration was performed in triplicate wells, and repeated twice for the MTT assay.

The photocytotoxicity of samples was assessed by a similar protocol. In general, HeLa cells (3 × 10^3^ per well) were incubated in 96-well plates for 24 h prior to their exposure to the drugs. The cells were treated with the samples in the dark overnight. Afterwards, the cells were exposed to a 50 W xenon lamp fitted with a heat-isolation filter and a 500 nm long-pass filter for 10 min. The fluence rate was 6 mW/cm^2^. The cell viability was determined by the MTT reduction assay.

#### Bioimaging Applications of CQDs@PtPor

Cellular imaging was evaluated using a confocal laser scanning microscope. HeLa cells (5 × 10^4^ cells per well) were seeded in 6-well culture plates and allowed to adhere for 12 h. The cells were then treated with CQDs@PtPor (0.25 mg/mL) at 37 °C for 1 h. After that, the supernatant was carefully removed and the cells were washed three times with PBS. Subsequently, the slides were mounted and observed by confocal microscope (Zeiss Laser Scanning Confocal Microscope; LSM7 DUO) using ZEN 2009 software (Carl Zeiss).

## Results

### Preparation of CQDs@PtPor

CQDs were prepared through a one-pot hydrothermal reaction according to the method described in literature [38], as shown in Scheme 1. The PtPor was synthesized through complexation of substituted transplatin with 5, 10, 15, 20-Tetra(4-pyridyl)porphyrin according to the reported method [37]. Since the PtPor molecule has four positive charges in the pyridine ring, which could bind on the surfaces of the negatively charged CQDs through an electrostatic interaction, yielding the desired CQDs@PtPor composite.

### Characterization of CQDs@PtPor

**Fig. 1:**
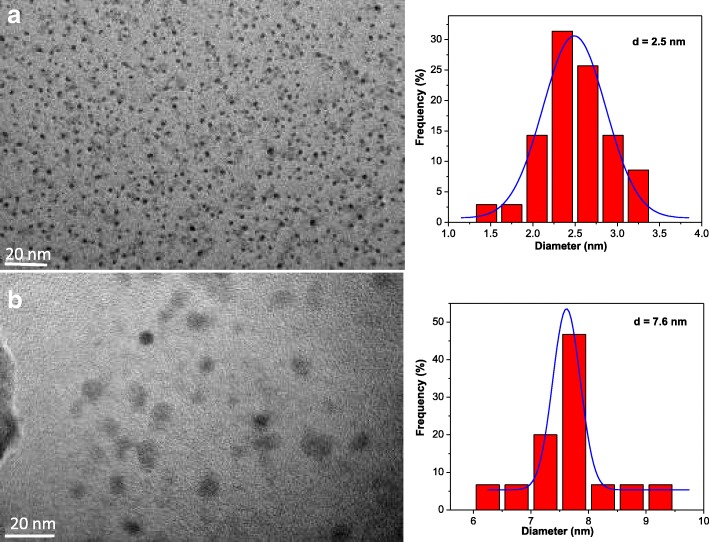
TEM images (left) and corresponding size distribution histograms (right) of **a** CQDs and **b** CQDs@PtPor

The transmission electron microscope (TEM) images (Fig. 1 left) show that the as-prepared CQDs and CQDs@PtPor are homogeneously distributed with uniform sizes. The particle size shown in Fig. 1 right is narrow (1–9 nm) and the average size, determined by histogram, is 2.5 and 7.6 nm for CQDs and CQDs@PtPor, respectively. The mean size of CQDs@PtPor is larger than that of CQDs, probably due to the adsorption of PtPor molecule on the surface of CQDs through an electrostatic interaction.

**Fig. 2:**
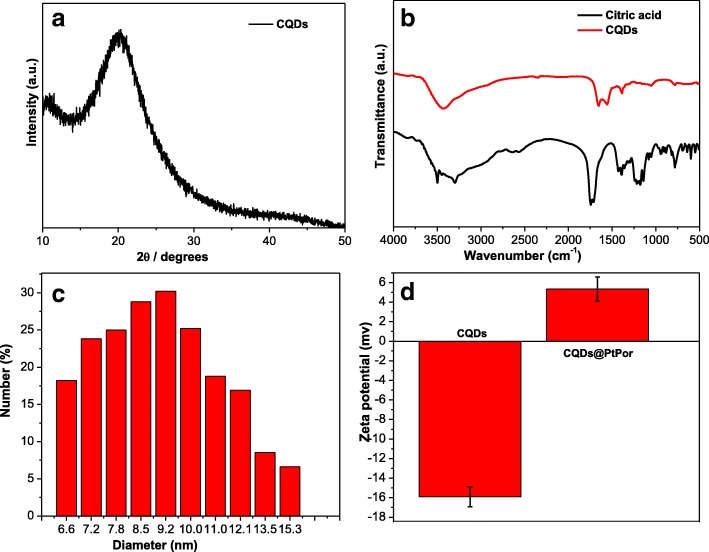
XRD pattern of CQDs (**a**). The FTIR spectrum of CQDs (**b**). Particle size distribution of CQDs@PtPor measured by dynamic light scattering (**c**). Zeta potential of CQDs and CQDs@PtPor (**d**)

**Fig. 3:**
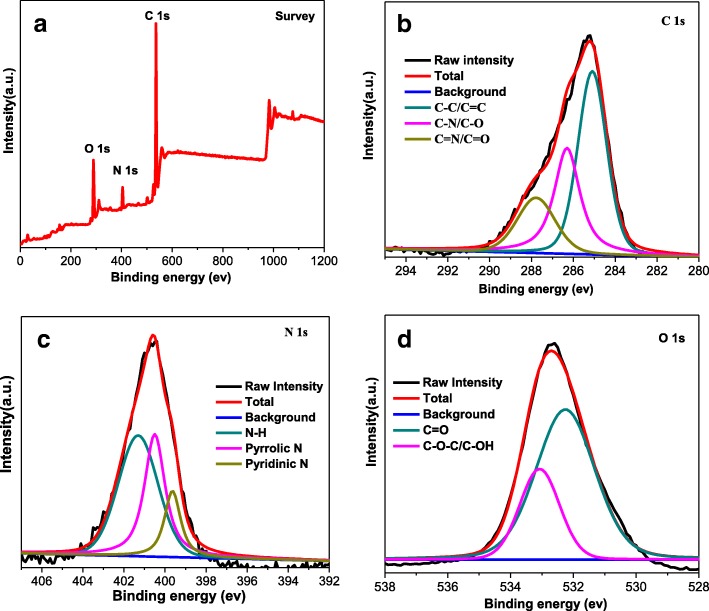
XPS survey spectrum (**a**), and C 1s (**b**), N 1s (**c**), and O 1s (**d**) high-resolution XPS spectra of CQDs

Figure 2a shows the X-ray diffraction (XRD) pattern of the as-synthesized CQDs. The broad XRD peak of CQDs appears around 23°, indicating the disordered structure of CQDs [39]. The functional groups of CQDs were characterized by FTIR spectroscopy. As shown in Fig. 2b, the broad peaks from 3000 to 3500 cm^−1^ are attributed to O-H and N-H stretching vibrations, indicating the presence of hydroxyl and amino groups. The peaks at 1150 and 1230 cm^−1^ are attributed to the C-O and C-N stretching vibrations, respectively. The amide bond is confirmed by the typical peaks at 1678 and 1392 cm^−1^, attributing to the vibrations of amide’s C=O and C-N, respectively. Finally, the peak at 1600 cm^−1^ is identified as C=C/C=N bond. Comparing with the FTIR results of citric acid, the CQDs did not display any significant characteristic absorption of citric acid (CA), indicating that CA should be mostly carbonized during the process of hydrolysis. Besides, a new sharp peak at 1700 cm^−1^, ascribed to the amide bond was found, indicating that ethylenediamine should be functionalized on the surface of CQDs through the -CONH- linkage. The mean diameter and particle size distribution of CQDs and CQDs@PtPor were determined by DLS measurement (Additional file 1: Figure S1 and Fig. 2c). As shown in Fig. 2c, the mean size of CQDs@PtPor is about 9.2 nm, which is consistent with the result from that of TEM test. The zeta potential measurement was further conducted to confirm the conjugation between CQDs and PtPor. As shown in Fig. 3d, the zeta potential of free CQDs is − 15.6 mV, due to the negative charges on the surface. After conjugation with PtPor, the zeta potential of CQDs@PtPor composite was changed to 4.5 mV, indicating the successful coverage of CQDs by the PtPor molecules.

X-ray photoelectron spectroscopy (XPS) was performed to further investigate the chemical composition of CQDs (Fig. 3) and CQDs@PtPor (Additional file 1: Figure S2). The survey spectrum of CQDs in Fig. 3a indicates the existence elements on the surface are C, N, and O, with the related signals at 535, 402, and 283 eV, respectively [40]. The C 1s signal shown in Fig. 3b has three distinct peaks at 284.4 eV, 286.3 eV, and 288.2 eV, which are assigned to the C-C bond, C-O bond, and C=O bond, respectively. The high-resolution XPS N 1s shown in Fig. 3c is fitted with three peaks, with binding energies at 395.3, 399.1, and 402.2 eV, corresponding to the pyridine-like N, pyrrolic N, and quaternary N, respectively [41]. The deconvolution of O 1s exhibited the C-O and O-H peaks (Fig. 3d), indicating the existence of large carboxylic groups on the surface of CQDs.

### Photophysical Properties of CQDs@PtPor

**Fig. 4:**
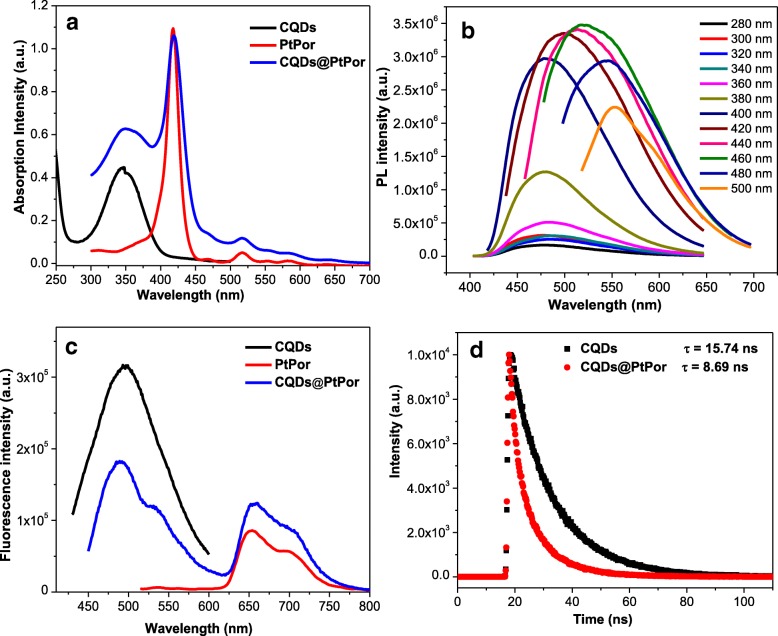
UV-Vis absorption (**a**) and fluorescence (**b**) spectra of CQDs, PtPor, and CQDs@PtPor. Fluorescence spectra (**c**) of CQDs with different excitation wavelengths. Fluorescence decays (**d**) of CQDs and CQDs@PtPor. The concentration of samples: CQDs (5 μg/mL), CQDs@PtPor (5 μg/mL, 3 μg/mL), and PtPor (3 μg/mL)

The UV-Vis absorption and fluorescence spectra were run to investigate the photophysical properties of the composite. As shown in Fig. 4a, the CQDs@PtPor composite showed the characteristic peaks from CQDs and porphyrin. For example, a significant absorption peak around 360 nm was probably assigned to the n → π* transition from CQDs [42], while the peaks around at 425 nm, 520 nm, and 580 nm were attributed to the soret and Q bands of porphyrin, respectively. The aqueous solution of CQDs shows blue emission under the irradiation of a 365-nm ultraviolet (UV) lamp. Besides, the CQDs exhibited the excitation-dependent PL behavior, where the emission peak shifted from 460 to 552 nm when the excitation wavelength changed from 280 to 500 nm, as shown in Fig. 4b. The fluorescence quantum yield of the as-prepared CQDs was 36% with quinine sulphate as a reference.

## Discussion

The fluorescence resonance energy transfer (FRET) effect in CQDs@PtPor composite could be investigated through comparing the fluorescence intensity of CQDs@PtPor with CQDs and PtPor. The fluorescence spectra and intensities of CQDs, PtPor, and CQDs@PtPor with the same concentration were measured under the excitation of 360 nm (Fig. 4c). Since the CQDs showed very strong absorption at 360 nm (Fig. 4a) with the PL quantum yield as high as 36%, it emits very strong fluorescence. On the contrary, as the absorption of PtPor at 360 nm is very low, and its PL quantum yield is less than 1%, the PtPor exhibits very weak emission. Remarkably, the intensity of blue emission (500 nm) in CQDs@PtPor decreased obviously compared with free CQDs, while the red emission (660 nm) is significantly enhanced relative to that of PtPor alone, indicating the efficient energy transfer in CQDs@PtPor composite. The fluorescence lifetime of CQDs in CQDs@PtPor composite decreased relative to that of free CQDs, as shown in Fig. 4d. Such an evident decrease in the donor lifetime further indicates the effective resonance energy transfer from CQDs to PtPor in the CQDs@PtPor composite.

**Fig. 5:**
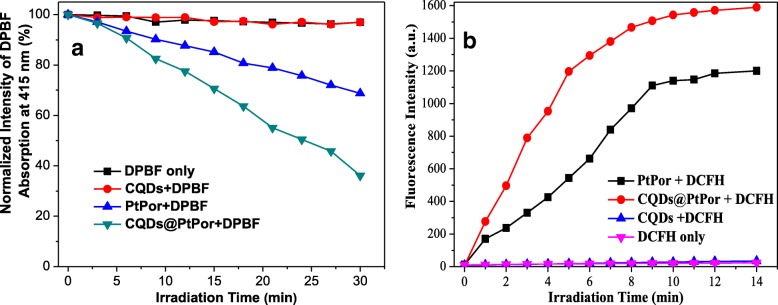
Singlet oxygen generation of CQDs, PtPor, and CQDs@PtPor from DPBF method (**a**) and DCFH method (**b**)

Since the singlet oxygen production is a key factor in PDT, the ^1^O_2_ generation was determined by a chemical method using 1,3-diphenylisobenzofuran (DPBF) as the ^1^O_2_ scavenger. In general, the absorption intensity of DPBF will decrease gradually in the presence of singlet oxygen. Therefore, the decrease rate of the absorption intensity of DPBF can be used to evaluate the relative yield of singlet oxygen. In this experiment, CQDs (5 mg/mL), PtPor (5 mg/mL), or CQDs@PtPor (5 mg/mL) was mixed with DPBF (10 mM), respectively, followed by the irradiation with xenon lamp. As shown in Fig. 5a, after the addition of CQDs, the absorption of DPBF did not show any change with the prolongation of irradiation time, indicating that CQDs had no significant singlet oxygen production. Besides, the CQDs@PtPor composite exhibited a very obvious degradation to DPBF, which is much higher than that of PtPor, indicating that the ^1^O_2_ yield of porphyrin could be enhanced under the role of CQDs. Meanwhile, the production of ^1^O_2_ was further quantified using the dichlorofluorescein (DCFH) reagent. The green fluorescence (λ_em_ = 525 nm) of DCFH is known to increase quantitatively when it reacts with ^1^O_2_ generated from the photosensitizers. As shown in Fig. 5b, the CQDs@PtPor composite showed higher efficiency of ^1^O_2_ production than that of pure PtPor. This result is highly consistent with that obtained by the DPBF method.

**Fig. 6:**
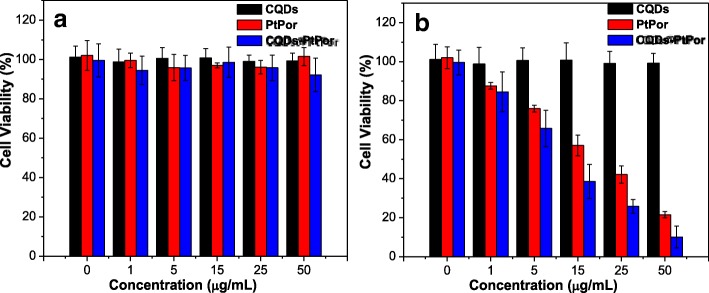
Dark cytotoxicity (**a**) and photocytotoxicity (**b**) of CQDs, PtPor, and CQDs@PtPor at different concentrations

The cytotoxicity of CQDs, PtPor, and CQDs@PtPor upon HeLa cells was tested through the methylthiazolyltetrazolium (MTT) assay. As shown in Fig. 6a, all three samples exhibited the negligible cytotoxicity against HeLa cells after treatment for 24 h in the dark. Over 90% of cancer cells was still alive with their concentration increased to 50 μg/mL, suggesting that all three samples had no adverse effect on cancer cells in the dark. Moreover, the photocytotoxicity of three samples was further evaluated using a similar method. As shown in Fig. 6b, after treatment of cancer cells with CQDs@PtPor for 24 h followed by light irradiation, the cell viability decreased gradually with the increase of sample concentration. When the concentration of CQDs@PtPor was 50 μg/mL, the survival rate of cancer cells was only 8%, which was apparently lower than that of PtPor alone (18%) and CQDs (90%). That is, the CQDs@PtPor composite exhibited stronger therapeutic efficacy than that of PtPor alone, suggesting that CQDs@PtPor is advantageous over the conventional formulation, which is probably ascribed to the enhanced efficiency of singlet oxygen generation of PtPor by CQDs.

**Fig. 7:**
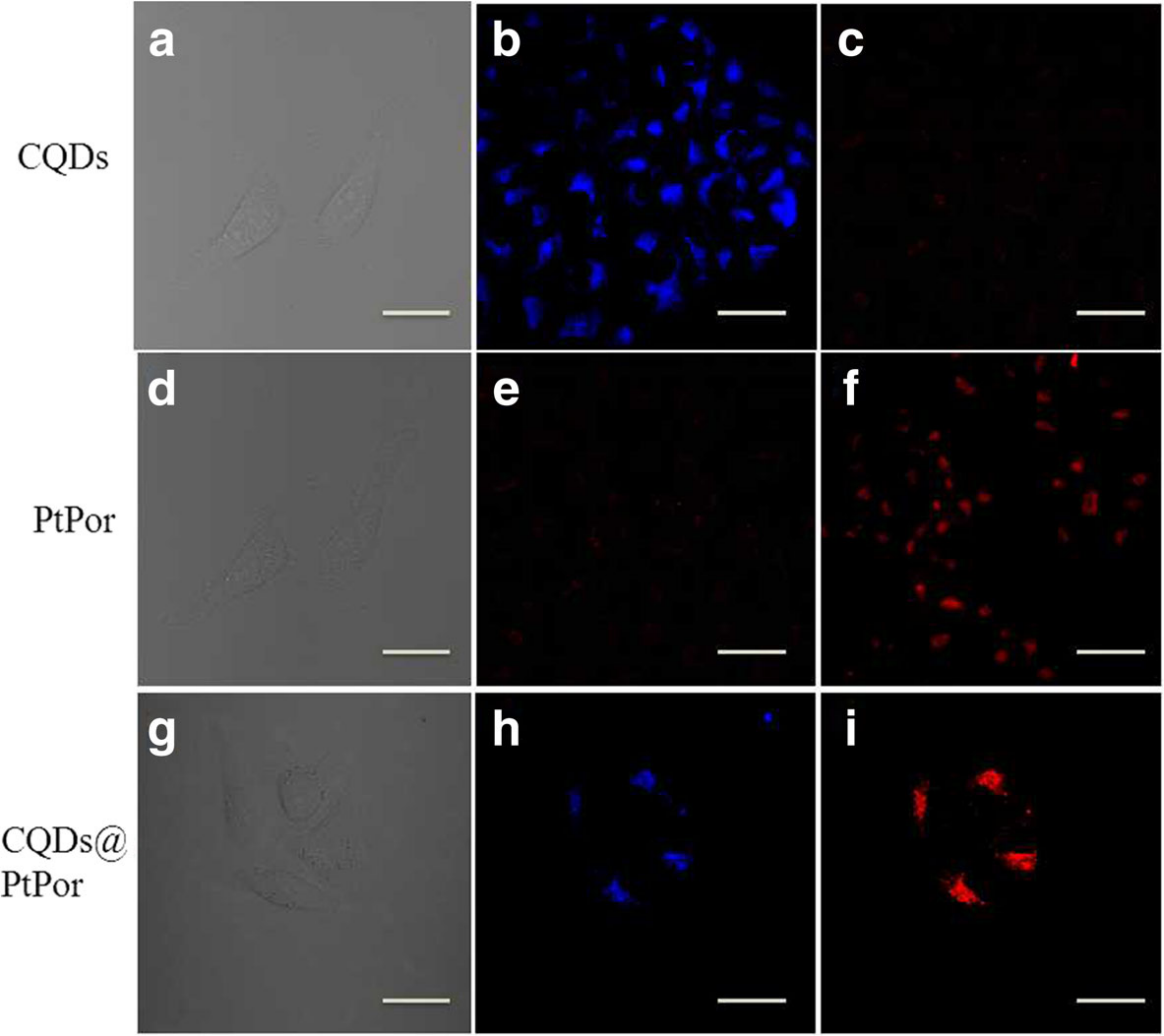
The confocal fluorescence microscopy images of HeLa cells under 405 nm excitation after treatment with 50 μg/mL of pure CQDs (**a**–**c**), PtPor (**d**–**f**), and CQDs@PtPor (**g**–**i**) for 24 h. **a**, **d**, **g** Bright field. **b**, **e**, **h** The CQDs imaging channel, detected at the wavelength region of 410–450 nm. **c**, **f**, **i** The PtPor imaging channel, detected with the 590 nm long pass region; (scale bar = 20 μm)

The cellular uptake of pure CQDs, PtPor, and CQDs@PtPor was studied using a confocal laser scanning microscope under excitation of a 405-nm laser. As shown in Fig. 7, the CQDs@PtPor composite mainly distributed in the cytoplasm of HeLa cells. Besides, the blue fluorescence imaging from CQDs is almost overlapped with the red emission from that of PtPor in CQDs@PtPor composite, indicating that the CQDs and PtPor remained in binding state after the CQDs@PtPor composite entered into cells. These results verify that the CQDs@PtPor composite is stable in the cellular environment and could still perform fluorescence resonance energy transfer in cells.

## Conclusions

A new theranostic nanoagent (CQDs@PtPor) was successfully designed and developed through the electrostatic interaction between the tetraplatinated porphyrin complex (PtPor) and the negatively charged CQDs. The as-prepared CQDs@PtPor composite exhibited high water dispersibility, good stability and biocompatibility, and enhanced photosensitizer fluorescence detection. The PDT effect of CQDs@PtPor was significantly enhanced relative to that of PtPor alone, suggesting that CQDs@PtPor is advantageous over the conventional formulation due to the enhanced efficiency of ^1^O_2_ generation of PtPor by CQDs. Thus, this CQDs-based nanoagent displayed enhanced therapeutic efficacy upon cancer cells as well as low side effects in vitro, showing great potential for applications in the clinic to treat patients with cancer in the near future.

## Additional file

Additional file 1: Supporting Information. (DOCX 151 kb)

## Abbreviations

CA: Citric acid
CQDs: Carbon quantum dots
DPBF: 1,3-Diphenylisobenzofuran
EDA: Ethylenediamine
MTT: 3-(4,5-Dimethylthiazol-2-yl)-2,5-diphenyltetrazolium bromide
PDT: Photodynamic therapy
PSs: Photosensitizers
TEM: Transmission electron microscope
UV: Ultraviolet
XPS: X-ray photoelectron spectroscopy
XRD: X-ray diffraction
