# Correction to: Carbon Dots @ Platinum Porphyrin Composite as Theranostic Nanoagent for Efficient Photodynamic Cancer Therapy

**DOI:** 10.1186/s11671-018-2838-1

**Published:** 2019-01-09

**Authors:** Fengshou Wu, Liangliang Yue, Huifang Su, Kai Wang, Lixia Yang, Xunjin Zhu

**Affiliations:** 1 Key Laboratory for Green Chemical Process of the Ministry of Education, School of Chemical Engineering and Pharmacy, Wuhan Institute of Technology, Wuhan, 430205 People’s Republic of China; 2 Department of Orthopaedics, The First Affiliated Hospital of Zhengzhou University, Zhengzhou, 450052 People’s Republic of China; 3 Department of Chemistry, Hong Kong Baptist University, Waterloo Road, Hong Kong, People’s Republic of China

**Correction to: Nanoscale Research Letters**

**https://doi.org/10.1186/s11671-018-2761-5**

Following publication of the original article [1], it was flagged that Fig. 4 and Fig. 5 in the article were (incorrectly) formatted with a yellow highlighting of the background of the figures.

This formatting error has since been corrected; however we apologize for this processing error.
