# Supplementary material for: Carbon Dots @ Platinum Porphyrin Composite as Theranostic Nanoagent for Efficient Photodynamic Cancer Therapy
Source: Nanoscale Res Lett. 2018 Nov 8;13:357. doi: 10.1186/s11671-018-2761-5 (PMC6223393; doi:10.1186/s11671-018-2761-5)
Supplement: Supplementary file 1 — Supporting Information. (DOCX 151 kb) [file 11671_2018_2761_MOESM1_ESM.docx]

**Supporting Information**

**Carbon Dots @ Platinum Porphyrin Composite as Theranostic Nanoagent for Efficient Photodynamic Cancer Therapy**

Fengshou Wu,^*1^ Liangliang Yue,^1^ Huifang Su,^2^ Kai Wang,^1^ Lixia Yang,^1^ Xunjin Zhu^3^

^a^ Key Laboratory for Green Chemical Process of the Ministry of Education, School of Chemical Engineering and Pharmacy, Wuhan Institute of Technology, Wuhan 430205, P. R. China. E-mail: fswu@wit.edu.cn.

^b^ Department of Orthopaedics, The First Affiliated Hospital of Zhengzhou University, Zhengzhou, 450052, P. R. China.

^c^ Hubei Callaborative Innovation Center for Advanced Organic Chemical Materials, College of Chemistry and Chemical Engineering, Hubei University, Wuhan, P. R. China.

^d^ Department of Chemistry, Hong Kong Baptist University, Waterloo Road, Hong Kong, P. R. China. E-mail: xjzhu@hkbu.edu.hk.

**Experimental Sections**

**Materials and Instruments**

Trans-platinum diammine dichloride (transplatin) was purchased from Aladdin^®^. DPBF was obtained from Sigma-Aldrich. All the solvents were purchased from Tianjin Fu Chen Chemical Reagents. The other chemicals were purchased from Sinopharm Chemical Reagent Co., Ltd and used as received. Fourier Transform Infrared (FT-IR) spectra were recorded on a Bruker Vertex 70 spectrometer. X-ray photoelectron spectroscopy (XPS) was performed using an SKL-12 spectrometer modified with a VG CLAM 4 multichannel hemispherical analyzer. Photoluminescence (PL) spectra were recorded on a Perkin-Elmer LS 55 spectrofluorometer. The electronic absorption spectra in the UV/Vis region were recorded with a Hewlett Packard 8453 UV/Vis spectrophotometer. The transmission electron microscopic (TEM) analyses were performed on an FEI-TECNAI G2 S-TWIN TEM, operating at an acceleration voltage of 200 kV. X-ray powder diffraction measurements were investigated using a Bruker D8 Advance diffractometer through nonmonochromatic Cu Kα X-ray at 40 kV, 40 mA.

**Synthesis**

Synthesis of [trans-PtCl(NH_3_)_2_]_4_-5,10,15,20-tetra(4-pyridyl)-porphyrin nitrate (PtPor)

Transplatin (0.193 mmol, 58 mg) and silver nitrate (0.193 mmol, 33 mg) were dissolved in 5 mL of DMF. After string for 24 hours, the white silver chloride formed was removed from the resulted turbid solution through the centrifugation to acquire the clear solution, which was then added to the suspension of 5,10,15,20-Tetra(4-pyridyl)porphyrin (0.487 mmol, 30 mg) in 3 mL DMF. After stirring at 50 °C for 48 hours, the mixture was cooled down to room temperature. 10 mL diethyl ether was added to get the red precipitate, which was then washed with methanol, dichloromethane and diethylether. Finally, the sample was dried under vacuum to acquire 81 mg product. Yield 86%. ^1^H NMR (400 MHz; DMSO-d_6_): δ 9.45 (d, 8H), 9.14 (s, 8H), 8.52 (m-pyridyl, d, 8H), 4.70 (NH_3_, s, 24H), -3.04 (s, 2H); MS (ESI): m/z = 1209 [M-3(NO_3_)-2{PtCl(NH_3_)_2_}]^+^, 1074 [M-4(NO_3_)-2{PtCl(NH_3_)_2_}-2NH_3_-Cl-2H]^+^, 883 [M-4(NO_3_)-3{PtCl(NH_3_)_2_}]^+^, 866 [M-4(NO_3_)-3{PtCl(NH_3_)_2_}-NH_3_]^+^, 812 [M-4(NO_3_)-3{PtCl(NH_3_)_2_}-Cl-2(NH_3_)]^+^, 574 [M-4(NO_3_)-2{PtCl(NH_3_)_2_}]^2+^.

Preparation of the CQDs

Generally, citric acid (0.45 g) and ethylenediamine (500 μL) was dissolved in DI-water (10 mL). Then the solution was transferred to a poly (tetrafluoroethylene) (Teflon)-lined autoclave (30 mL) and heated at 200 ºC for 5 h. After the reaction, the reactors were cooled to room temperature by water or naturally. The product, which was brown-black and transparent, was subjected to dialysis in order to obtain the CDs.

Preparation of the CQDs@PtPor conjugate

The PtPor molecule, bearing 4 positive charges in pyridine ring, can bind on the surfaces of the negatively charged CQDs through an electrostatic interaction to obtain the CQDs@PtPor conjugate. In general, 20 mg PtPor dissolved in 10 mL DMF was added slowly into the CQDs suspension (5 mg CQDs dissolved in 10 mL H_2_O) under sonication. After stirring at room temperature for 24 hours, the solution was purified in a centrifuge for 30 min to remove agglomerated particles, and then dialyzed against DI water for two days. The aqueous solution of CQDs@PtPor was lyophilized at 4 °C to yield the desired product.

The calculation of quantum yields of CQDs

The quantum yield of CQDs was measured with quinine sulfate as the reference (0.1 M H_2_SO_4_ aqueous solution, fluorescent quantum yield ∼ 54%) by the following equation:

φ=φ_st_(I/I_st_)(η/η_st_)^2^

Where Φ is fuorescence quantum yield, I is the slope of curves, and η is the refractive index of solvent. The subscript “st” refers to the reference of known quantum yield (quinine sulfate in 0.1 M H_2_SO_4_). The absorption was kept below 0.1 at the excitation wavelength of 360 nm to minimize reabsorption.

**Singlet oxygen generation**

A solution of the sample and 3-diphenylisobenzofuran were irradiated in a glass cuvette (3 mL), at room temperature. The absorption decay of DPBF at 415 nm was measured at irradiation intervals of 3 min up to 30 min. The production of singlet oxygen was evaluated qualitatively through the DPBF, a singlet oxygen quencher. The percentage of the DPBF absorption decay, proportional to the production of ^1^O_2_, was assessed by the difference between the initial absorbance and the absorbance after a given period of irradiation. Each experiment was repeated 3 times

Cytotoxicity assay of CQDs, PtPor, and CQDs@PtPor

Human cervical carcinoma (HeLa) cells were cultured in DMEM (Dulbecco’s Modified Eagle’s Medium) supplemented with 5% FCS (Fetal Calf Serum), 100 U/mL penicillin, 100 μg/mL streptomycin at 37 ^°^C and 6% CO_2_. The MTT viability assay was performed according to a standard method. In brief, HeLa cells (3×10^3^/ well) were seeded in 96-well plates for 24 hours prior to exposure to drugs. The cells were treated with samples overnight in the dark. The cytotoxicity was determined by the MTT reduction assay. The cell monolayers were rinsed twice with phosphate-buffered saline (PBS) and then incubated with 50 μL MTT solution (0.5 mg/ mL) at 37^°^C for 3 hours. After the media were removed, 100 μL of DMSO was added. The solution was shook for 30 minutes to dissolve the formed formazan crystals in living cells. The absorbance was measured at dual wavelength, 540 nm and 690 nm, on a Labsystem Multiskan microplate reader (Merck Eurolab, Switzerland). Each dosed concentration was performed in triplicate wells, and repeated twice for the MTT assay.

The photocytotoxicity of samples was assessed by a similar protocol. In general, HeLa cells (3×10^3^/ well) were incubated in 96-well plates for 24 hours prior to exposure to drugs. The cells were treated with samples in the dark overnight. Afterwards, the cell was exposure to a xenon lamp. Cell viability was determined by the MTT reduction assay.

Bioimaging applications of CQDs@PtPor

Cellular imaging was evaluated using a confocal laser scanning microscope. HeLa cells (5 x 10^4^ cells per well) were seeded in 6-well culture plates and allowed to adhere for 12 h. The cells were then treated with CQDs@PtPor (0.25 mg/mL) at 37 ^o^C for 1 h. After that, the supernatant was carefully removed and the cells were washed three times with PBS. Subsequently, the slides were mounted and observed by confocal microscope (Zeiss Laser Scanning Confocal Microscope; LSM7 DUO) using ZEN 2009 software (Carl Zeiss)

Figure S1. Particle size distribution of CQDs measured by dynamic light scattering (DLS).

Figure S2. XPS spectra of CQDs@PtPor
